# Supplementary material for: High-dose corticosteroid pulse therapy increases the survival rate in COVID-19 patients at risk of hyper-inflammatory response
Source: PLoS One. 2021 Jan 28;16(1):e0243964. doi: 10.1371/journal.pone.0243964 (PMC7842890; doi:10.1371/journal.pone.0243964)
Supplement: S1 Table — P-value and false discovery rate (FDR) of 30-day time-course analysis between survivors and non-survivors are included. Markers with statistically significant changes are highlighted in grey. (DOCX) [file pone.0243964.s001.docx]

**S1 Table.** All forty-five laboratory makers tested. P-value and false discovery rate (FDR) of 30-day time-course analysis between survivors and non-survivors are included. Markers with statistically significant changes are highlighted in grey.

| **Laboratory Markers** | **P value** | **FDR** | **Unit of Measurement** | **Normal Values** |
| --- | --- | --- | --- | --- |
| Urea | 1.27E-63 | 5.86E-62 | mg/dL | [ 10 - 50 ] |
| Lactate dehydrogenase | 9.45E-52 | 2.17E-50 | U/L | [ 0 - 250 ] |
| C-reactive protein | 1.19E-49 | 1.82E-48 | mg/L | [ 0.0 - 5.0 ] |
| Haemoglobin | 3.00E-36 | 3.44E-35 | g/dL | [ 13.0 - 18.0 ] |
| Absolute neutrophil count | 6.73E-30 | 6.20E-29 | x 10^3/μL | [ 2.00 - 7.50 ] |
| Platelets | 8.18E-30 | 6.27E-29 | x 10^3/μL | [ 130 - 450 ] |
| Glomerular filtration rate | 6.86E-24 | 4.51E-23 | mL/min |  |
| Gamma-glutamyl transferase | 1.53E-23 | 8.79E-23 | U/L | [ 8 - 61 ] |
| Computed Fibrinogen | 3.51E-21 | 1.79E-20 | mg/dL | [ 150 - 400 ] |
| D-dimer | 4.92E-18 | 2.26E-17 | ng/mL | [ 0 - 500 ] |
| Interleukin 6 | 1.03E-17 | 4.30E-17 | pg/mL | [ 0.00 - 7.00 ] |
| Percentage Eosinophil | 1.95E-15 | 7.46E-15 | % | [ 0.00 - 6.00 ] |
| Mean Corpuscular Volume | 5.38E-13 | 1.90E-12 | fL | [ 82.0 - 95.0 ] |
| Glucose | 1.17E-11 | 3.83E-11 | mg/dL | [ 74 - 106 ] |
| Total basophils | 2.76E-10 | 8.48E-10 | x 10^3/μL | [ 0.00 - 0.10 ] |
| Alanine transaminase | 3.16E-10 | 9.09E-10 | U/L | [ 0 - 41 ] |
| Ferritin | 5.93E-08 | 1.61E-07 | ng/mL | [ 30.0 - 400.0 ] |
| Procalcitonin | 3.88E-05 | 9.91E-05 | ng/mL | 0.0 - 0.05 |
| Total protein content | 9.25E-05 | 2.24E-04 | g/dL | [ 6.4 - 8.3 ] |
| Partial Thromboplastin Time (seconds) | 2.08E-04 | 4.78E-04 | seg | [ 25.0 - 37.0 ] |
| Triglyceride | 2.69E-04 | 5.90E-04 | mg/dL | [ 30 - 150 ] |
| Aspartate transaminase | 5.49E-04 | 1.15E-03 | U/L | [ 0 - 40 ] |
| Mean corpuscular haemoglobin | 7.19E-04 | 1.44E-03 | pg | [ 27.0 - 32.0 ] |
| Concentration of mean corpuscular haemoglobin | 8.94E-04 | 1.71E-03 | g/dL | [ 32.0 - 36.0 ] |
| Troponin T (high sensitivity) | 1.34E-03 | 2.47E-03 | ng/L | [ 0.0 - 14.0 ] |
| Total monocytes | 1.52E-03 | 2.68E-03 | x 10^3/μL | [ 0.00 - 1.00 ] |
| Red blood cells | 6.75E-03 | 1.15E-02 | x 10^6/μL | [ 4.00 - 5.50 ] |
| Prothrombin time | 1.20E-02 | 1.97E-02 | seg | [ 11.0 - 14.0 ] |
| Fibrinogen | 2.46E-02 | 3.88E-02 | mg/dL | [ 150 - 400 ] |
| Total lymphocyte count | 2.53E-02 | 3.88E-02 | x 10^3/μL | [ 1.00 - 4.00 ] |
| International normalized ratio | 3.76E-02 | 5.58E-02 | INR | [ 0.80 - 1.20 ] |
| Total Bilirubin | 7.69E-02 | 1.11E-01 | mg/dL | [ 0.1 - 1 ] |
| Creatine kinase | 1.18E-01 | 1.51E-01 | U/L | [ 39 - 308 ] |
| Leucocyte | 1.17E-01 | 1.51E-01 | x 10^3/μL | [ 3.70 - 9.70 ] |
| Leucocyte percentage | 1.18E-01 | 1.51E-01 | % | [ 20.00 - 45.00 ] |
| Monocyte percentage | 1.14E-01 | 1.51E-01 | % | [ 2.00 - 10.00 ] |
| Prothrombin time (percentage) | 2.07E-01 | 2.57E-01 | % | [ 70 - 120 ] |
| Albumin | 2.96E-01 | 3.58E-01 | g/dL | [ 3.5 - 5.2 ] |
| Haematocrit | 5.20E-01 | 5.98E-01 | % | [ 40.0 - 54.0 ] |
| Neutrophils percentage | 5.14E-01 | 5.98E-01 | % | [ 40.00 - 75.00 ] |
| Mean platelet volume | 5.93E-01 | 6.65E-01 | fL | [ 7.0 - 11.0 ] |
| Dispersion de hemateins (volume) | 6.99E-01 | 7.66E-01 | % | [ 11.0 - 16.0 ] |
| Basophils percentage | 7.95E-01 | 8.31E-01 | % | [ 0.00 - 1.00 ] |
| Creatinine | 9.17E-01 | 9.37E-01 | mg/dL | [ 0.70 - 1.20 ] |
| Total Eosinophils | 9.92E-01 | 9.92E-01 | x 10^3/μL | [ 0.00 - 0.30 ] |
