# Supplementary material for: High-dose corticosteroid pulse therapy increases the survival rate in COVID-19 patients at risk of hyper-inflammatory response
Source: PLoS One. 2021 Jan 28;16(1):e0243964. doi: 10.1371/journal.pone.0243964 (PMC7842890; doi:10.1371/journal.pone.0243964)
Supplement: Attachment — Submitted filename: Response to reviewers.docx [file pone.0243964.s002.docx]

Response to reviewers:

Dear reviewers,

First of all, thank you for the time dedicated and the proposals you have made on the “*High-Dose Corticosteroid Pulse Therapy increases the survival rate in COVID-19 patients at risk of hyper-inflammatory response*” by Lopez-Zuñiga *et al*. Below we respond to your suggestions.

Yours sincerely,

The Autors.

**Reviewer #1: A good job. This is a well-designed and conducted prospective observational study. The conclusion is HDCPT can decrease the mortality of COVID 19 patients with signs of cytokine storm.**

**I have a few minor concerns:**

1. **Who took the low dose corticosteroid therapy?**

We thank the reviewer pointing out this missing information, we have now added this to the methods as follows:

“*Low dose corticosteroid therapy was administered to patients who had had a bronchospasm, following standard clinical guidelines.”*

**2. "16 patients received HDCPT due to their critical clinical status." Can you explain it in detail?**

We thank the reviewer for requesting this clarification, these details have been added to the methods section as follows:

“*These 16 patients received high dose corticosteroids pulse therapy because they developed severe respiratory failure and did not respond to the standard COVID-19 clinical practice at that time, including pharmacological (hydroxychloroquine, azithromycin and lopinavir-ritonavir) and physical interventions (i.e. postural changes).*”

**3. Last, did all the patients have laboratory tests every day until they discharged or died? No matter they stayed in ICU or not? Please provide details.**

These details have now been added to the text:

“*All the patients who were in ICU had laboratory tests every 24h. Patients outside of ICU had laboratory tests every 48h unless showing worsening symptoms (in these cases they were tested every 24h).*”

**Reviewer #2: For this study the authors collected data in an observational and prospective fashion for confirmed or suspected COVID-19 cases. They computed and followed up various laboratory markers to distinguish survivors and non-survivors, thus to describe a group of patients with poor prognosis whom may require high-dose pulse steroid therapy (>1.5mg/kg/d methylprednisolone or equivalent dose of dexamethasone) for 2-5 days. Several factors were compared retrospectively between survivors and non-survivors.
This is well written manuscript however suffers from the fact that it is not randomized controlled trial. As the authors also admitted there many confounding factors which may have affected their results. There have been already several randomized controlled trials including RECOVERY trial and and at least one meta-analysis (.doi:10.1001/jama.2020.17023) looking for the effect of steroids in critically ill COVID-19 patients. RECOVERY trial tested a lower dose of dexamethasone (i.e. 6 mg/d) than the current study albeit longer duration. Thus, this study may have had a conclusion saying that high doses of steroids (i.e. 1,5 mg/kg/d) for short courses may have an impact on mortality. However, the design of this study would not allow such conclusions.
My further comments include the following:**

1) **The authors refer a "cytokine storm" throughout the text which has been severely criticised in the literature (doi:10.1001/jamainternmed.2020.3313). Perhaps "hyper-inflammatory response" is a better term which has also been used alternatively.**

We thank the reviewer for his/her suggestion. We have now replaced all the instances in the text.

2) **The authors defined pre-set criteria for high dose steroid use, but less than one-third of patients were given the drug. In addition, 16 patients without these criteria received it. This is a clear bias. Since the study is not randomized and controlled, various imbalances may possibly exist in groups receiving and non-receiving high dose steroids.**

We thank the reviewer for querying further into this. We agree with the reviewer and acknowledge the limitations of the study design. Overall, we have not observed any major biases in any of the variables measured and our analyses are carried out using multivariate approaches. However, we have amended the discussion and abstract to reflect these limitations, and they are further acknowledged in our conclusions.

At the time this study was performed, some experts hypothesized the presence of a hyperinflammatory syndrome, which could point to a beneficial role for corticosteroids in treating COVID-19. However, the World Health Organisation warned against the use of corticosteroids in patients with SARS-Cov-2. Because of this, some doctors did not want to treat their patients without scientific evidence and therefore some patients were not treated with corticosteroids, despite having similar clinical profiles. In addition, 16 patients were given corticosteroids due to the critical status despite not meeting the pre-defined criteria. Our pre-defined criteria was set following the guidelines used for another other pro-inflammatory syndrome (macrophage activation syndrome) and our analysis of pro-inflammatory markers suggests that this criteria could be re-defined for COVID-19 patients.

3) **Defining high-dose of steroids is also arbitrary. In general high dose pulse steroid may usually refer dose equaling 250 mg or higher methylprednisolone given with short duration of infusion.**

We thank the reviewer for querying the dose. To set the high-dose of corticosteroids we followed guidelines from studies in other autoimmune diseases in which they found that, above methylprednisolone doses of 125 mg there were no clinical differences. Recent studies in COVID-19 patients have also corroborated this (Callejas *et al* 10.1016/j.medcli.2020.04.018 and Ruiz-Irastorza *et al* 10.1371/journal.pone.0239401).

We have added this to the discussion as follows:

*“In this study we define high dose corticosteroids pulses as doses of at least 125 mg of methylprednisolone or dexamethasone equivalent. Previous studies in COVID-19 patients did not found clinical differences between methylprednisolone doses above 125 mg [*Callejas *et al* and Ruiz-Irastorza *et al*]*”*

**4) The duration of steroid therapy is defined as 2-5 days which lacks standardization and the effects of therapy may differ between those who received 2 days vs 5 days.**

We thank the reviewer for querying this. The treatment duration has now been clarified in the text as follows:

*“The standard high dose corticosteroid pulse therapy duration was 3 days. In some patients who did not improve after 3 days, the treatment was extended to 5 days. In two patients, the treatment was shortened to 2 days due to the significant recovery observed.”*
