# Supplementary material for: High-dose corticosteroid pulse therapy increases the survival rate in COVID-19 patients at risk of hyper-inflammatory response
Source: PLoS One. 2021 Jan 28;16(1):e0243964. doi: 10.1371/journal.pone.0243964 (PMC7842890; doi:10.1371/journal.pone.0243964)
Supplement: Attachment — Submitted filename: Response to reviewers_v2docx.docx [file pone.0243964.s003.docx]

Dear reviewers,

First of all, thank you for the time dedicated and the proposals you have made on the “*High-Dose Corticosteroid Pulse Therapy increases the survival rate in COVID-19 patients at risk of hyper-inflammatory response*” by Lopez-Zuñiga *et al*. Below we respond to your suggestions.

Yours sincerely,

The Authors.

***Authors observed the increased survival in COVID-19 patients with HDCPT and proposed a potential COVID-19 specific criteria to diagnose the development of COVID-19 cytokine storms. It is a very interesting study, for which could clinically direct doctors to use HDCPT for the treatment of COVID-19 patients. However, there still have several questions need to address.***

1. ***The current results have shown that HDCPT is effective. it is better to divide the patients into mild/moderate,  severe and critical categories and observe survival rates after HDCPT based on the guidelines from NIH or Europe (such as*** [***https://www.covid19treatmentguidelines.nih.gov/overview/clinical-presentation/***](https://www.covid19treatmentguidelines.nih.gov/overview/clinical-presentation/)***).***

We thank the reviewer for his/her suggestion to inspect the results by disease category. According to the NIH guidelines, out of the 318 patients included in our study, 20 patients were moderate (6.3%). The rest of the patients were all severe or critical (n=238, 93.4%). Unfortunately, we cannot distinguish between severe and critical patients, since we do not have data for respiratory or multi organ failure, and not all critical patients were admitted to intensive care due to overcrowding at the height of the pandemic. We have now included these details in the revised manuscript.

In addition, we have recomputed our multivariate analysis after removing moderate patients, the results are very similar:

**Analysis without moderate patients (n=298)**

**Analysis with all patients (presented in the manuscript, n=318)**

2. ***Virus clearance time should be addressed the study to value the adverse effects with HDCPT patients.***

We agree with the reviewer that it would be very interesting to examine the effects of HDCPT on virus clearance time. Unfortunately, during the time of the study we did not collect any information to tackle this issue. In the revised version of the manuscript have added virus clearance time to the discussion as one key factor to cover in future studies as following:

Other studies have shown that corticosteroid therapy does not affect virus clearance time [10.1038/s41598-020-70387-2]. Unfortunately, in our study we did not carry out a follow up quantify viral clearance. It would be interesting to assess whether HDCPT has an impact on virus clearance times and we hope that future studies can shed light on this topic.

3. ***In figure 2, the laboratory tests also need to carried out in severity categories, which will make the differences more significant***

Similarly to the previous point, we have reanalysed the data removing all the moderate patients. The results are quite similar and in some cases the results are slightly more significant.

**Analysis without moderate patients (n=298)**

**Analysis with all patients (presented in the manuscript, n=318)**

4. ***Several studies had reported that hydroxychloroquine have no benefit on COVID-19 patients, which seems controversial with the result. Please discuss this point in the discussion section.***

We thank the reviewer for suggesting a further discussion on the effect of hydroxycholoquine as it is a controversial topic in this COVID-19. We have now added this to the discussion as follows:

The role of hydroxycholoquine in COVID-19 remains controversial. In our study we only found a marginal association between the use of hydroxycholoquine and an increase in survival rate. Although this study was not designed to assess the role of hydroxycholoquine in survival rates, this marginal association could be in line with what had been reported in previous studies in which they found that hydroxychloroquine was effective at inhibiting SARS-CoV-2 in vitro [^[1]^]. On the contrary, randomized clinical trials such as RECOVERY [^[2]^] and the one carried out by Cavalcanti *et al* [^[3]^] did not found an increase in survival rates in COVID-19 patients. However, the clinical study carried out by Cavalcanti only looked at mild and/or moderate COVID-19 patients and the RECOVERY study did not study severe hospitalized patients. To date, there is not enough evidence to suggest that hydroxycholoquine is effective at increasing the survival of COVID-19 patients and more studies are needed to dissect the effects of hydroxycholoquine in COVID-19.

1. Yao X, Ye F, Zhang M,  [Cheng Cui](https://pubmed.ncbi.nlm.nih.gov/?term=Cui+C&cauthor_id=32150618), [Baoying Huang](https://pubmed.ncbi.nlm.nih.gov/?term=Huang+B&cauthor_id=32150618), [Peihua Niu](https://pubmed.ncbi.nlm.nih.gov/?term=Niu+P&cauthor_id=32150618) et al. In Vitro Antiviral Activity and Projection of Optimized Dosing Design of Hydroxychloroquine for the Treatment of Severe Acute Respiratory Syndrome Coronavirus 2 (SARS-CoV-2) [published online ahead of print, 2020 Mar 9]. Clin Infect Dis. 2020;ciaa237. doi:10.1093/cid/ciaa23]
2. RECOVERY Collaborative Group, Horby P, Mafham M, et al. Effect of Hydroxychloroquine in Hospitalized Patients with Covid-19 [published online ahead of print, 2020 Oct 8]. *N Engl J Med*. 2020;NEJMoa2022926. doi:10.1056/NEJMoa2022926
3. Cavalcanti AB, Zampieri FG, Rosa RG, et al. Hydroxychloroquine with or with- out azithromycin in mild-to-moderate Covid-19. N Engl J Med. DOI: 10.1056/ NEJMoa2019014.
